# Tn-Seq Analysis Identifies Genes Important for Yersinia pestis Adherence during Primary Pneumonic Plague

**DOI:** 10.1128/mSphere.00715-20

**Published:** 2020-08-05

**Authors:** Kara R. Eichelberger, Victoria E. Sepúlveda, John Ford, Sara R. Selitsky, Piotr A. Mieczkowski, Joel S. Parker, William E. Goldman

**Affiliations:** a Department of Microbiology and Immunology, University of North Carolina at Chapel Hill, Chapel Hill, North Carolina, USA; b Lineberger Comprehensive Cancer Center, University of North Carolina, Chapel Hill, North Carolina, USA; c Department of Genetics, University of North Carolina, Chapel Hill, North Carolina, USA; University of Kentucky

**Keywords:** Tn-seq, *Yersinia pestis*, adherence, lung, plague, pneumonic plague

## Abstract

Following inhalation, Yersinia pestis rapidly colonizes the lung to establish infection during primary pneumonic plague. Although several adhesins have been identified in *Yersinia* spp., the factors mediating early Y. pestis adherence in the lung remain unknown. To identify genes important for Y. pestis adherence during primary pneumonic plague, we used transposon insertion sequencing (Tn-seq). Wild-type and capsule mutant (Δ*caf1*) Y. pestis transposon mutant libraries were serially passaged *in vivo* to enrich for nonadherent mutants in the lung using a mouse model of primary pneumonic plague. Sequencing of the passaged libraries revealed six mutants that were significantly enriched in both the wild-type and Δ*caf1* Y. pestis backgrounds. The enriched mutants had insertions in genes that encode transcriptional regulators, chaperones, an endoribonuclease, and YPO3903, a hypothetical protein. Using single-strain infections and a transcriptional analysis, we identified a significant role for *YPO3903* in Y. pestis adherence in the lung and showed that YPO3903 regulated transcript levels of *psaA,* which encodes a fimbria previously implicated in Y. pestis adherence *in vitro*. Deletion of *psaA* had a minor effect on Y. pestis adherence in the lung, suggesting that YPO3903 regulates other adhesins in addition to *psaA*. By enriching for mutations in genes that regulate the expression or assembly of multiple genes or proteins, we obtained screen results indicating that there may be not just one dominant adhesin but rather several factors that contribute to early Y. pestis adherence during primary pneumonic plague.

**IMPORTANCE** Colonization of the lung by Yersinia pestis is a critical first step in establishing infection during primary pneumonic plague, a disease characterized by high lethality. However, the mechanisms by which Y. pestis adheres in the lung after inhalation remain elusive. Here, we used Tn-seq to identify Y. pestis genes important for adherence early during primary pneumonic plague. Our mutant enrichment strategy resulted in the identification of genes important for regulation and assembly of genes and proteins rather than adhesin genes themselves. These results reveal that there may be multiple Y. pestis adhesins or redundancy among adhesins. Identifying the adhesins regulated by the genes identified in our enrichment screen may reveal novel therapeutic targets for preventing Y. pestis adherence and the subsequent development of pneumonic plague.

## Abstract (precis)

Colonization of the lung by Yersinia pestis is a critical first step in establishing infection during primary pneumonic plague, a disease characterized by high lethality. However, the mechanisms by which Y. pestis adheres in the lung after inhalation remain elusive. Here, we used Tn-seq to identify Y. pestis genes important for adherence early during primary pneumonic plague. Our mutant enrichment strategy resulted in the identification of genes important for regulation and assembly of genes and proteins rather than adhesin genes themselves. These results reveal that there may be multiple Y. pestis adhesins or redundancy among adhesins. Identifying the adhesins regulated by the genes identified in our enrichment screen may reveal novel therapeutic targets for preventing Y. pestis adherence and the subsequent development of pneumonic plague.

## INTRODUCTION

Inhalation of aerosolized droplets containing Yersinia pestis causes primary pneumonic plague, which is the most severe manifestation of plague. Once in the lung, Y. pestis establishes an anti-inflammatory environment that is permissive for rapid bacterial proliferation and leads to severe pulmonary inflammation (1, 2). Adherence to host cells is a critical first step during bacterial pathogenesis (3), and Y. pestis likely requires adhesins to mediate attachment to airway cells for colonization of the lungs during primary pneumonic plague. The early events of primary pneumonic plague, particularly how Y. pestis adheres to cells in the small airways, are incompletely understood.

Two of the best-characterized adhesins in the genus *Yersinia* are YadA and invasin. Yersinia pseudotuberculosis and Yersinia enterocolitica utilize invasin and YadA to establish infection in the small intestine (4). Invasin binds β1 integrins on M cells, promoting internalization of the enteropathogenic *Yersinia* organisms (5). YadA has more diverse functions, including mediating attachment to epithelial cells and extracellular matrix proteins as well as promoting persistence of Y. enterocolitica in the Peyer’s patches (6, 7). However, both YadA and invasin are absent in Y. pestis, due to an inactivating mutation in *yadA* and an insertional element in *inv* (8, 9). Therefore, other factors must be involved in facilitating Y. pestis adherence to host cells and colonization, particularly during primary pneumonic plague.

Using various cell culture infection models, four surface structures displayed by Y. pestis have been implicated in adherence: Pla, Ail, PsaA, and the F1 antigen. Y. pestis *pla* mutants are attenuated in primary pneumonic plague models of disease (10). The plasminogen activator protease, or Pla, has effects on adherence that are independent of its proteolytic activity (11). Pla binds laminin present in extracellular matrices and promotes invasion of HeLa cells (12, 13). Pla also facilitates Y. pestis type III secretion system (T3SS) translocation into alveolar macrophages (14, 15). However, Y. pestis *pla* mutants adhere to epithelial cell lines as well as wild-type Y. pestis, suggesting that Pla does not function as a dominant adhesin to the respiratory epithelium (16). In contrast, the attachment invasion locus, *ail*, has been proposed to encode a major adhesin in *Yersinia*. The *ail* gene is present and functional in all three human-pathogenic *Yersinia* and confers serum resistance (17–19). However, its effect on adherence varies among *Yersinia* species. Ail mediates host cell binding *in vitro* for Y. enterocolitica, but if introduced into nonpathogenic Y. enterocolitica species that do not contain the *ail* gene, it does not confer attachment or invasion (20). Deletion of *ail* in Y. pseudotuberculosis does not alter adhesion to host cells (18). In Y. pestis, adherence to epithelial cell lines *in vitro* is reduced only when *ail* is deleted in combination with other surface protein-encoding genes or when the bacteria are grown at an environmental temperature of 28°C (16, 21).

PsaA, or the pH 6 antigen, is a fimbria-like structure displayed on the cell surface of Y. pestis at 37°C and pH 6 (22). Y. pestis Δ*psaA* is less adherent to epithelial lines *in vitro* and also inhibits phagocytosis by macrophages when the bacteria are grown under inducing conditions of low pH and 37°C (23, 24). However, when *psaA* is deleted in a strain of Y. pestis lacking the F1 antigen, the bacteria are just as adherent as wild-type cells, suggesting the presence of other unknown adhesins (23). The F1 antigen, which is encoded by the *caf1* gene on the pMT1 plasmid, is a large polymer that forms an antiphagocytic capsule-like structure surrounding Y. pestis at 37°C (25–27). Y. pestis Δ*caf1* is more adherent to epithelial cell lines, and its deletion reveals the presence of large fimbria-like structures that extend from the cell surface (23, 28). Thus, expression of F1 antigen on the bacterial surface appears to mask or block the effect of other Y. pestis adhesins. It is unknown if Ail, PsaA, or the F1 antigen affects Y. pestis adherence in the lung during primary pneumonic plague.

Despite the identification and characterization of several proteins in the outer membrane of Y. pestis, no single factor has emerged as the dominant Y. pestis adhesin (21, 23, 29). Therefore, we used transposon insertion sequencing (Tn-seq) as an unbiased approach to identifying genes important for Y. pestis adherence during primary pneumonic plague. We performed *in vivo* serial enrichment using our mouse model of primary pneumonic plague to screen wild-type and Δ*caf1* Y. pestis transposon mutant libraries for nonadherent mutants in the lung. Comparison of the identities of nonadherent mutants in Y. pestis wild-type and Δ*caf1* libraries indicated that there were six independent mutants significantly enriched in both strain backgrounds. These mutants had insertions in *YPO3904* and *iscR*, which encode transcriptional regulators, in *tig* and *clpX*, which encode chaperones, in *rnc*, which encodes an endoribonuclease, and in *YPO3903*, which encodes a hypothetical protein. We demonstrated a significant role for YPO3903 in Y. pestis adherence in the lung and in regulating transcript levels of *psaA*, the deletion of which had a minor effect on Y. pestis adherence in the lung. The enrichment of mutants that likely have altered gene expression and levels of multiple proteins suggests functional redundancy or the presence of multiple adhesins in Y. pestis.

## RESULTS

### Yersinia pestis genes important for adherence in the lung are unknown.

**FIG 1:**
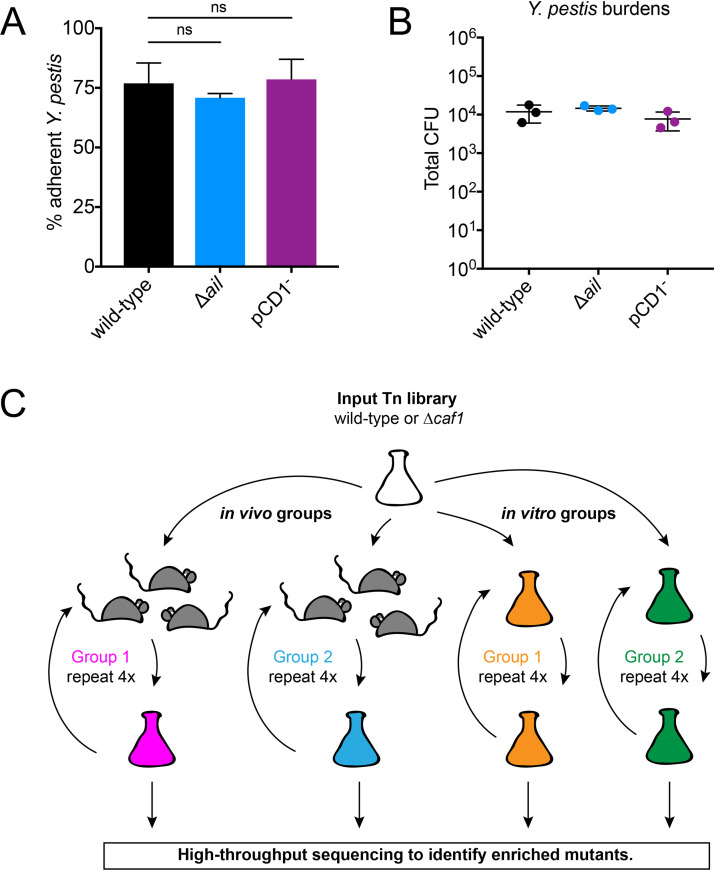
The Yersinia pestis genes important for adherence in the lung are unknown. Mice were inoculated intranasally with 1 × 10^4^ CFU of the Y. pestis wild-type, Δ*ail*, or pCD1^−^ strains, and bronchoalveolar lavage was performed at 2 hpi. CFU were enumerated in both the bronchoalveolar lavage fluid (BALF) and the lung. (A) Percent adherence was calculated for each strain by determining the proportion of Y. pestis in the lung compared to total CFU in both the BALF and the lung. Significance was determined by one-way ANOVA with Tukey’s multiple correction. ns, not significant. (B) Total Y. pestis CFU enumerated in both the lung and the BALF. Data are representative of two independent experiments with 3 mice per group and are means and SD. (C) Schematic depicting the screen method for serial enrichment of nonadherent Y. pestis transposon mutants in both wild-type and Δ*caf1* strains. The input library was inoculated into 2 groups of 3 mice each or 2 *in vitro* culture flasks. At 2 hpi, bronchoalveolar lavage was performed on the mice to collect nonadherent Y. pestis mutants and a portion of the *in vitro* cultures were removed. These mutants were then used to inoculate new groups of mice and *in vitro* cultures, repeating the *in vivo* enrichment or *in vitro* controls, and this process was repeated for a total of four times to collect mutants for high-throughput sequencing to determine the identity of the enriched mutants.

The earliest events of primary pneumonic plague, particularly how Y. pestis interacts with and adheres to cells in the small airways, are unknown. To characterize the initial adherence of Y. pestis in the lung, we used our mouse model of primary pneumonic plague to determine the proportion of adherent bacteria early after inoculation (2). Mice were inoculated intranasally with 1 × 10^4^ CFU Y. pestis CO92 (wild type), and 2 h postinoculation (hpi), we performed a bronchoalveolar lavage of the lungs to remove any nonadherent Y. pestis and enumerated the proportion of Y. pestis that remained in the lung. We observed that approximately 75% of Y. pestis was adherent in the lung 2 h after inoculation (Fig. 1A, black bar).

The attachment invasion locus (Ail) has been implicated in adherence for Y. enterocolitica and Y. pestis to various cell lines, and a Y. pestis *ail* mutant exhibits reduced virulence following intranasal inoculation (16, 20, 30). Therefore, *ail* may play a role in adherence of Y. pestis in the lung early after inoculation. Additionally, it was recently shown that the Y. pestis type III secretion system (T3SS) needle tip protein, LcrV, binds the *N*-formylpeptide receptor (FPR1) on immune cells for delivery of T3SS effectors (31). It is therefore possible that T3SS binding to host FPR1 mediates Y. pestis adherence in the lung. To test the role of *ail* and the T3SS in Y. pestis adherence early during primary pneumonic plague, we inoculated mice intranasally with 1 × 10^4^ CFU Δ*ail* Y. pestis or pCD1^−^ Y. pestis, which lacks the plasmid encoding the T3SS needle apparatus and effectors (25). At 2 hpi, we performed a bronchoalveolar lavage and calculated the percentage of adherent Y. pestis for each strain. Approximately 75% of Y. pestis Δ*ail* and pCD1^−^ was adherent in the lung, similar to the levels of adherence for wild-type Y. pestis (Fig. 1A). Total bacterial burdens in the lung for the three Y. pestis strains were also similar (∼10^4^ CFU) at 2 hpi (Fig. 1B). Thus, despite the putative role of *ail* in Y. pestis adherence *in vitro* and LcrV binding to FPR1, our data suggest that these surface structures do not individually play a major role in mediating Y. pestis adherence in the lung early during primary pneumonic plague.

To identify genes important for Y. pestis adherence in the lung, we took an unbiased genetic approach using Tn-seq to screen comprehensive libraries of Y. pestis mutants for genes required for adherence. We predicted that mutants with transposon insertions in genes important for adherence in the lung would be enriched in the bronchoalveolar lavage fluid (BALF) collected from mice following intranasal inoculation with a Y. pestis transposon library. To enhance the enrichment of nonadherent mutants, we also created a comprehensive library of transposon insertional mutants in a Y. pestis Δ*caf1* strain in addition to the wild-type Y. pestis mutant library. The *caf1* gene encodes the F1 antigen, which forms a large capsule-like structure around Y. pestis at 37°C (26, 27). The presence of the F1 antigen on the surface of Y. pestis masks surface structures and adherence *in vitro* (23, 28). Therefore, the Δ*caf1* strain background may provide greater enrichment of nonadherent mutants without the masking effect of the F1 antigen.

A schematic depicting our enrichment strategy is shown in Fig. 1C. Briefly, we generated transposon input libraries of approximately 233,000 mutants for wild-type Y. pestis and approximately 208,000 mutants for Y. pestis Δ*caf1*. We then inoculated two groups of three mice each intranasally with either 1 × 10^6^ CFU Y. pestis wild-type transposon mutants or 5 × 10^6^ Y. pestis Δ*caf1* transposon mutants. Using a higher inoculum than our standard dose of 1 × 10^4^ CFU ensured delivery of all transposon mutants to the lung. At the same time, a portion of each input library was inoculated into two replicate *in vitro* liquid cultures and grown with shaking at 37°C for *in vitro* growth controls. At 2 hpi, we collected BALF (containing Y. pestis mutants with adherence defects) from mice and a portion of each *in vitro* culture, and we plated these groups of mutants separately on selective media. Portions of these enriched mutants were then used to start liquid cultures for the next round of infection, keeping the two biological replicates for the *in vivo* and *in vitro* groups separate. This enrichment protocol was repeated three more times for a total number of four rounds of enrichment *in vivo* and *in vitro*. After each round of enrichment, genomic DNA was isolated from each group of Y. pestis mutants for sequencing of the transposon junctions to identify the enriched mutants.

### Enrichment of less-adherent Y. pestis mutants after serial passaging of transposon libraries.

**FIG 2:**
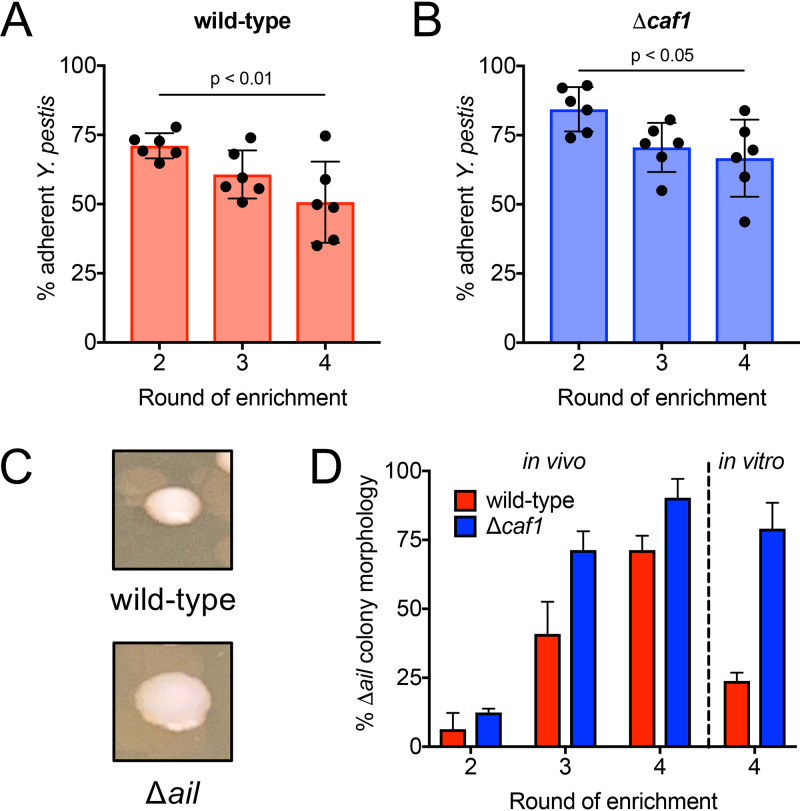
Enrichment of less-adherent Y. pestis mutants after serial passaging of transposon libraries. Mice were inoculated intranasally with 1 × 10^6^ CFU of the Y. pestis wild-type transposon library or 5 × 10^6^ CFU of the Y. pestis Δ*caf1* transposon library. Bronchoalveolar lavage was performed at 2 hpi, and CFU were enumerated in both the bronchoalveolar lavage fluid (BALF) and the lung. Percent adherence was calculated after two, three, and four rounds of *in vivo* enrichment by determining the proportion of Y. pestis transposon mutants in the lung compared to the total CFU of transposon mutants in both the BALF and the lung for wild-type (A) and Δ*caf1* (B) backgrounds. *P* values (A and B) were determined by one-way ANOVA with Tukey’s multiple correction. (C) Colony morphology of a wild-type Y. pestis colony and a Δ*ail* Y. pestis colony, both in the pCD1^−^ strain background, grown at 26°C for 2 days on BHI agar. (D) The proportion of colonies in the BALF with a Δ*ail-*like morphology at two, three, and four rounds of enrichment *in vivo* and four rounds of enrichment *in vitro* for both wild-type (red bars) and Δ*caf1* libraries (blue bars). Data are means and SD.

During our Tn-seq screen, we calculated the proportion of adherent bacteria after each round of enrichment *in vivo* for both Y. pestis wild-type and Δ*caf1* groups. The proportion of adherent wild-type Y. pestis insertional mutants in the lung decreased from 70% after two rounds of enrichment to ∼60% by three rounds and ∼50% by four rounds of enrichment (Fig. 2A). There was also a reduction in adherence for Y. pestis mutants in the Δ*caf1* background over four rounds of enrichment. There was approximately 85% adherence after two rounds of selection, and this decreased to ∼70% adherence by three rounds and ∼65% adherence by four rounds of enrichment (Fig. 2B). Together, these data indicate that there was significant enrichment of nonadherent mutants by serially passaging both the Y. pestis wild-type and Δ*caf1* transposon libraries through the lungs of mice. Additionally, the Y. pestis Δ*caf1* mutants were more adherent in the lung than the wild-type Y. pestis mutants at each round of selection, supporting previous observations that the F1 antigen masks Y. pestis adherence factors.

Correlated with the enrichment of nonadherent mutants after each *in vivo* passage of the transposon libraries, we also observed an increase in the proportion of an altered colony morphology for Y. pestis mutants collected in the BALF after each round of enrichment. The colonies were larger with a less-defined border compared to those of wild-type Y. pestis, which is indicative of an *ail* mutant (Fig. 2C) (21). For the nonadherent mutants in the wild-type background, approximately 6% of the colonies recovered in the BALF had the Δ*ail-*like colony morphology after two rounds of enrichment, which increased to 40% after three rounds and 70% after four rounds of enrichment (Fig. 2D, red bars). The enrichment for Δ*ail-*like mutants was stronger in the Δ*caf1* background, with 10% of nonadherent mutants having the Δ*ail-*like colony morphology after two rounds, increasing to 70% by three rounds and 90% by four rounds of enrichment (Fig. 2D, blue bars). However, we also observed enrichment of Δ*ail-*like mutants in the *in vitro* control groups. After four rounds of enrichment *in vitro*, ∼25% of the colonies in the wild-type *in vitro* controls had a Δ*ail-*like colony morphology, whereas nearly 75% of colonies in the Δ*caf1* background had a Δ*ail-*like morphology (Fig. 2D). The *in vitro* enrichment suggests that Δ*ail* mutants may have a competitive growth advantage during the *in vitro* culture steps between inoculations that contributed to their enrichment during the *in vivo* screen, particularly in the Δ*caf1* background. Due to the large proportion of Δ*ail-*like colonies (and thus likely mutants with transposon insertions in *ail* or genes that regulate *ail*) saturating the mutant pools after four rounds of enrichment, we performed high-throughput sequencing and subsequent analysis of the pools of nonadherent mutants collected after two and three rounds of selection.

### Significant enrichment of mutations in genes involved in the regulation and assembly of macromolecules.

We performed high-throughput sequencing using the MiSeq platform, sequencing the transposon junction to identify which mutants were enriched by the screen. We determined that approximately 25% of all TA sites in the genome contained a transposon insertion in the Δ*caf1* input library, and 77% of annotated genes in the genome had at least one TA site disrupted. For the wild-type input library, approximately 37% of all TA sites in the genome contained a transposon insertion, and 83% of annotated genes had at least one TA site disrupted. Considering that about 10 to 15% of Y. pestis annotated genes were calculated to be essential (32), our input libraries had relatively good coverage of the genome. The number of unique reads for each gene was counted, upper quartile normalized, and log_2_ transformed. As there were two replicates for the *in vivo* and *in vitro* groups, we compared the similarity of the read count for each gene between the two replicates for each condition (Fig. S1). We observed a very strong correlation between the two replicates for each condition, with *R*^2^ values of >0.99 for every condition except Δ*caf1* 2 rounds *in vitro*, which has an *R*^2^ value of 0.94. This indicates a high level of reproducibility among the technical replicates in the screen. Thus, we averaged the replicate read count for each gene.

10.1128/mSphere.00715-20.1 FIG S1 High similarity among technical replicates in the enrichment screen. The normalized read count (log_2_) for each gene in the group 1 for each *in vivo* and *in vitro* sample was plotted against the normalized read count (log_2_) for the same gene in the group 2 for each sample. A linear regression with an *R*^2^ goodness-of-fit test was performed for each group. Download FIG S1, PDF file, 2.4 MB. Copyright © 2020 Eichelberger et al. 2020 Eichelberger et al. This content is distributed under the terms of the Creative Commons Attribution 4.0 International license.

**FIG 3:**
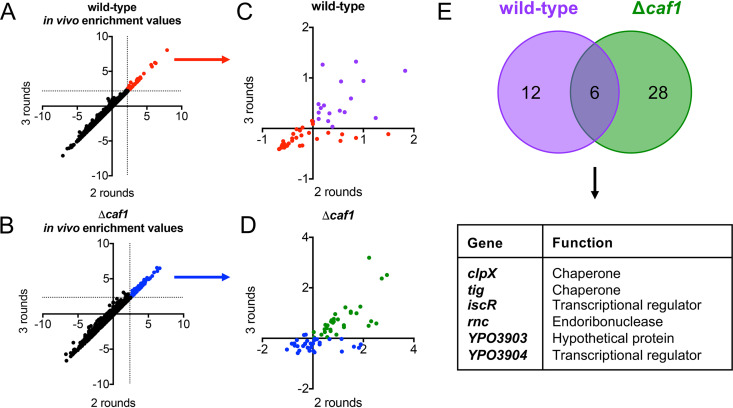
Significant enrichment of mutations in genes involved in the regulation and assembly of macromolecules. Libraries of the wild-type and Δ*caf1* transposon mutants were sequenced to identify the genes disrupted by the transposon insertion after two and three rounds of enrichment. Each gene was assigned an enrichment value, or the normalized log_2_ fold change of the number of unique reads mapping to the gene after enrichment relative to the number of reads mapping to that same gene in the input library. The *in vivo* enrichment values for each gene after 2 rounds of enrichment was plotted against the enrichment value for the same gene after 3 rounds of enrichment for wild-type (A) and Δ*caf1* (B) transposon libraries. Genes with enrichment values greater than 2 standard deviations (demarcated with dotted lines) from the mean after both 2 and 3 rounds of enrichment were highlighted. The corresponding *in vitro* enrichment values for each gene highlighted in panels A and B were subtracted from the *in vivo* enrichment values. The 18 genes with positive values are indicated in purple for the wild-type background (C) and 34 genes are indicated in green for the Δ*caf1* background (D). (E) Mutations in six genes were significantly enriched in both the wild-type and Δ*caf1* backgrounds.

We then calculated the enrichment value for each gene. The enrichment value equals the fold change in the read count for a given gene after two and three rounds of enrichment relative to the read count for that gene in the input library. The goal of our screen was to identify significant enrichment of mutants with insertions in genes involved in Y. pestis adherence, so we focused on genes with enrichment values that were greater than two standard deviations (SD) from the mean after both two rounds and three rounds of enrichment *in vivo*. In the wild-type background, there were 51 genes that had enrichment values >2 SD from the mean (Fig. 3A, red dots). In the Δ*caf1* background, there were 67 genes with enrichment values >2 SD from the mean (Fig. 3B, blue dots). For these significantly enriched mutants, we wanted to eliminate any that were enriched due to enhanced *in vitro* growth. To this end, we subtracted the *in vitro* enrichment values from the *in vivo* enrichment values for each significantly enriched gene (Fig. 3C and D). In the wild-type background, there were 18 genes that had greater enrichment values *in vivo* over *in vitro* (Fig. 3C, purple dots). In the Δ*caf1* background, there were 34 genes with greater enrichment values *in vivo* relative to *in vitro* (Fig. 3D, green dots). The identity and enrichment values for each of these genes are listed in Table 1.

**TABLE 1:** Identity of significantly enriched mutants^*a*^

Locus tag	Gene name	Description	Enrichment value			
			Wild type		Δ*caf1*	
			2 rounds	3 rounds	2 rounds	3 rounds
YPO2897	*iscR*	DNA-binding transcriptional regulator	2.33 (2.32)	2.39 (2.25)	3.01 (2.96)	2.96 (2.93)
YPO3156	*clpX*	ATP-dependent protease ATP-binding subunit	3.04 (2.93)	3.14 (2.95)	2.75 (2.71)	2.86 (2.61)
YPO3904	*hdfR*	Transcriptional regulator	3.79 (3.67)	3.96 (3.64)	2.61 (2.15)	2.69 (2.33)
YPO3903		Hypothetical protein	3.65 (3.43)	3.92 (3.47)	3.19 (2.46)	3.39 (2.72)
YPO2718	*rnc*	Ribonuclease III	3.56 (3.45)	3.75 (3.27)	2.99 (0.06)	3.44 (0.94)
YPO3158	*tig*	Trigger factor	5.62 (5.06)	5.95 (5.02)	4.15 (3.37)	4.32 (3.59)

YPO3424	*nadC*	Quinolinate phosphoribosyl-transferase	2.60 (2.29)	2.65 (2.51)	1.51 (1.55)	1.49 (1.60)
YPOs05	*micF*	sRNA	2.64 (1.40)	2.53 (2.33)	0.00 (0.00)	0.00 (0.00)
YPO3589	*pyrI*	Aspartate carbamoyltransferase	2.27 (1.94)	2.28 (1.98)	0.83 (−0.12)	1.14 (0.14)
YPO1817		Hypothetical protein	3.53 (2.90)	3.66 (3.34)	−0.33 (−0.57)	−0.06 (−0.12)
YPO0883		DNA binding protein	2.32 (1.79)	2.45 (2.10)	0.09 (0.17)	0.10 (0.23)
YPO3412	*speD*	*S*-Adenosylmethionine decarboxylase	2.38 (2.22)	2.55 (2.15)	0.53 (−0.43)	0.76 (0.01)
YPO3880		Hypothetical protein	2.24 (1.48)	2.55 (1.95)	0.34 (0.11)	0.38 (0.25)
YPO2756	*mnmC*	5-Methylaminomethyl-2-thiouridine methyltransferase	2.46 (1.46)	2.94 (2.00)	0.72 (0.84)	0.66 (0.65)
YPO0416	*waaQ*	Lipopolysaccharide core biosynthesis protein	7.90 (6.07)	8.05 (6.91)	1.10 (0.00)	1.00 (0.00)
YPt_47		tRNA Arg	2.67 (2.47)	3.43 (2.16)	−1.30 (−1.02)	−0.64 (−1.44)
YPO0650	*cca*	Multifunctional tRNA nucleotidyl transferase/2′3′-cyclic phosphodiesterase/2′nucleotidase/phosphatase	2.39 (1.53)	2.63 (1.31)	−0.13 (0.24)	−0.27 (0.09)
YPMT1.54		Hypothetical protein	4.06 (3.67)	4.16 (4.13)	0.06 (−1.69)	0.01 (0.21)

YPO3345		Hypothetical protein	−1.18 (−1.38)	−0.99 (−1.20)	4.23 (3.66)	4.09 (4.04)
YPO2503		Hypothetical protein	0.65 (0.41)	0.56 (0.82)	3.35 (3.01)	3.46 (3.31)
YPO1074a		Hypothetical protein	−0.09 (−0.28)	−0.12 (−0.17)	3.34 (2.86)	3.58 (3.33)
YPO1399		Hypothetical protein	−1.25 (−1.05)	−1.02 (−1.36)	4.36 (3.66)	4.59 (4.24)
YPO0135	*greB*	Transcription elongation factor	6.15 (6.16)	6.25 (6.11)	5.81 (5.26)	5.90 (5.51)
YPO1082	*dnaQ*	DNA polymerase III subunit epsilon	−1.91 (−1.76)	−1.86 (−1.87)	2.48 (1.25)	2.45 (2.01)
YPO1088		DNA-binding prophage protein	2.53 (2.23)	2.59 (2.67)	3.08 (0.87)	3.31 (2.82)
YPO0393		Hypothetical protein	0.23 (0.25)	0.19 (0.41)	2.92 (2.34)	3.15 (2.64)
YPt_15		Pseudogene	2.27 (2.82)	2.17 (2.50)	2.73 (1.50)	2.99 (2.47)
YPO0019	*engB*	Ribosome biogenesis GTP-binding protein	0.29 (0.84)	0.20 (0.52)	3.17 (2.68)	3.00 (2.47)
YPO1203a		Hypothetical protein	−0.69 (−0.25)	−0.65 (−0.60)	3.77 (1.25)	3.59 (3.00)
YPO1299	*fruK*	1-Phosphofructokinase	−1.89 (−1.59)	−2.00 (−1.65)	3.99 (1.68)	4.09 (3.41)
YPO3033		Hypothetical protein	6.29 (6.96)	6.16 (6.57)	6.22 (5.42)	6.56 (5.94)
YPO0901		Hypothetical protein	2.72 (3.32)	2.61 (2.98)	3.07 (2.24)	3.41 (2.74)
YPO2075	*rnd*	Ribonuclease D	2.61 (2.87)	2.63 (2.67)	3.13 (2.32)	3.26 (2.59)
YPOs02	*ssrS*	Noncoding RNA	4.12 (4.68)	4.02 (4.35)	3.41 (2.67)	3.56 (2.85)
YPO0060	*tdh*	l-Threonine 3-dehydrogenase	2.84 (3.46)	2.74 (3.09)	3.59 (2.60)	3.98 (3.23)
YPO2905	*ail*	Attachment invasion locus	3.96 (4.28)	4.02 (4.35)	3.93 (3.10)	4.34 (3.57)
YPO2235	*rnb*	Exoribonuclease II	4.57 (4.59)	4.69 (4.54)	4.82 (3.93)	5.14 (4.18)
YPO2621	*ubiF*	2-Octaprenyl-3-methyl-6-methoxy-1,4-benzoquinol hydroxylase	−1.90 (−1.49)	−1.93 (−1.73)	2.98 (1.25)	3.00 (2.01)
YPO1265		DEAD box helicase family protein	2.97 (3.59)	2.85 (3.23)	4.40 (2.90)	4.88 (3.87)
YPO1681	*cheZ*	Chemotaxis regulator	2.72 (3.31)	2.69 (2.94)	3.02 (1.51)	3.56 (2.50)
YPO2244	*rnfC*	Electron transport complex protein	0.24 (0.84)	0.13 (0.49)	3.34 (2.05)	3.70 (2.47)
YPO3587	*ptsO*	N-regulated PTS system (Npr) phosphohistidinoprotein-hexose phosphotransferase	1.19 (1.00)	1.74 (0.94)	2.75 (0.87)	2.59 (1.33)
YPO2591		Hypothetical protein	1.06 (1.40)	1.17 (1.11)	2.73 (0.00)	3.32 (0.95)
YPO0642		Hypothetical protein	1.70 (2.32)	1.58 (1.96)	4.27 (2.05)	4.52 (1.33)
YPO0480	*dapB*	Dihydrodipicolinate reductase	−1.08 (−0.74)	−1.18 (−0.84)	2.98 (2.05)	2.81 (2.66)
YPO1704	*proQ*	Solute/DNA competence effector	3.60 (4.16)	3.52 (3.83)	4.60 (3.90)	4.71 (4.43)

a Values are *in vivo* enrichment values for the genes that were >2 SD from the mean and had greater *in vivo* enrichment after two and three rounds of enrichment, with the corresponding *in vitro* values in parentheses. The first group of genes (no highlighting) fit these criteria for both the wild-type and the Δ*caf1* backgrounds, the second group (highlighted in light gray) fit these criteria only for the wild-type background, and the third group (highlighted in dark gray) fit these criteria only for the Δ*caf1* background.

Of the 18 genes identified in the wild-type background and the 34 genes identified in the Δ*caf1* background, six genes were shared between the two groups: *clpX*, *tig*, *iscR*, *rnc*, *YPO3903*, and *YPO3904* (Fig. 3C). This small overlap in the identity of the significantly enriched mutants is reflective of comparing the enrichment values for every gene in the wild-type background relative to the enrichment value for the same gene in Δ*caf1* background, where there was poor correlation at both two and three rounds of enrichment (Fig. S2). Because mutations in the six genes were significantly and independently enriched in both strain backgrounds, we hypothesized that these genes may play the greatest role in Y. pestis adherence in the lung. The six genes are all encoded on the Y. pestis chromosome, and none of these genes are annotated as adhesins, fimbriae, or other similar structures (33). Instead, these genes are all involved in either the regulation or assembly of macromolecules. The genes *tig*, which encodes trigger factor, and *clpX* both encode proteins that exhibit chaperone functions for structures that assemble in the bacterial cytoplasm (34, 35). The genes *iscR* and *YPO3904* encode transcriptional regulators, *rnc* encodes an endoribonuclease, and *YPO3903* encodes a hypothetical protein. By enriching for mutations in genes that are involved in regulating expression of multiple genes or assembly of multiple proteins, these data suggest that there may be several Y. pestis adhesins important for adherence during primary pneumonic plague.

10.1128/mSphere.00715-20.2 FIG S2 Low similarity of mutants enriched between wild-type and Δ*caf1* backgrounds. The *in vivo* enrichment values for each gene in the wild-type background was plotted against the *in vivo* enrichment value for the same gene in the Δ*caf1* background after two (A) and three (B) rounds of enrichment background. A linear regression with an *R*^2^ goodness-of-fit test was performed for each group. Download FIG S2, PDF file, 0.7 MB. Copyright © 2020 Eichelberger et al. 2020 Eichelberger et al. This content is distributed under the terms of the Creative Commons Attribution 4.0 International license.

### *YPO3903* contributes significantly to Y. pestis early adherence in the lung.

**FIG 4:**
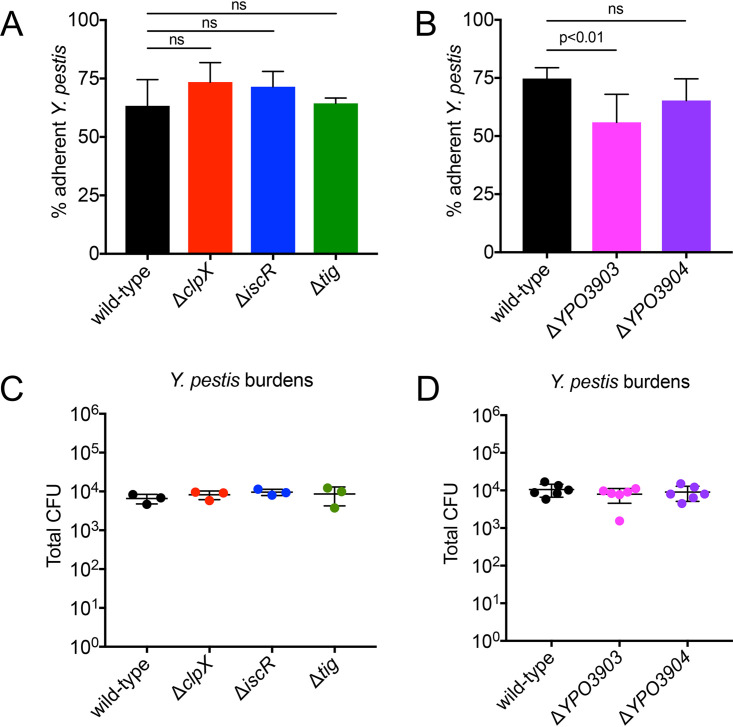
*YPO3903* contributes significantly to Y. pestis early adherence in the lung. Mice were inoculated intranasally with 1 × 10^4^ CFU of the Y. pestis wild-type, Δ*clpX*, Δ*iscR*, or Δ*tig* strains (A and C) and Y. pestis wild-type, Δ*YPO3903*, or Δ*YPO3904* strains (B and D), and bronchoalveolar lavage was performed at 2 hpi. CFU was enumerated in both the BALF and the lung. (A and B) Percent adherence was calculated for each strain by determining the proportion of Y. pestis in the lung compared to total CFU in both the BALF and the lung. (C and D) Total Y. pestis CFU enumerated in both the lung and the BALF. Data are representative of two independent experiments (A and C) or pooled from two independent experiments (B and D) with 3 mice per group. Data are means and SD. *P* values were determined by one-way ANOVA with Tukey’s multiple correction. ns, not significant.

To evaluate the effect on Y. pestis adherence for the six significantly enriched mutants in both the wild-type and Δ*caf1* backgrounds, we first created individual clean deletion strains for each gene, except *rnc*, in the wild-type Y. pestis background. RNase III, the endoribonuclease encoded by *rnc*, regulates rRNA processing, and *rnc* deletions result in global transcriptome changes that would be difficult to distinguish from other mRNAs that *rnc* may more specifically regulate (36–38). Therefore, due to the global, nonspecific effects of the *rnc* deletion, we chose not to further explore the effects of *rnc* in Y. pestis adherence. We inoculated mice intranasally with 1 × 10^4^ CFU of wild-type Y. pestis and the Δ*clpX*, Δ*iscR*, and Δ*tig* deletion strains. At 2 hpi, we performed a bronchoalveolar lavage to remove nonadherent bacteria and calculated the proportion of adherent Y. pestis. Y. pestis Δ*clpX*, Y. pestis Δ*iscR*, and Y. pestis Δ*tig* all had similar or even slightly higher levels of adherence (65 to 70% adherence) in the lung relative to the ∼65% adherence observed for wild-type Y. pestis (Fig. 4A). We next inoculated groups of mice with the single-deletion mutants Y. pestis Δ*YPO3903* and Y. pestis Δ*YPO3904* and compared the adherence at 2 hpi to that in mice inoculated with wild-type Y. pestis. Y. pestis Δ*YPO3903* had a significant reduction in adherence, with an average of 55% adherence compared to 75% for wild-type Y. pestis (Fig. 4B). Y. pestis Δ*YPO3904* had a small but statistically insignificant reduction in adherence relative to wild-type Y. pestis (Fig. 4B). Additionally, each Y. pestis strain had a similar total bacterial burden in the lung at 2 hpi, with ∼10^4^ CFU recovered (Fig. 4C and D). These data suggest that *YPO3903* plays a significant role in early Y. pestis adherence in the lung.

### *YPO3903* regulates *psaA* levels, which contributes to Y. pestis adherence in the lung.

Of the five mutations we analyzed for their role in Y. pestis adherence *in vivo*, only deletion of *YPO3903* had a significant effect on Y. pestis adherence in the lung. We explored the mechanism by which *YPO3903* could modulate adherence in the lung early after inoculation. *YPO3903* encodes a hypothetical protein that has 74% identity to the Escherichia coli protein YifE/MaoP (UniProt accession no. A0A384LBX8) (39). MaoP is conserved in *Enterobacteriaceae* and is involved in control of chromosome conformation and segregation (40). As chromosome organization could affect transcription, we hypothesized that the function of YPO3903 could be to regulate the expression of multiple genes involved in Y. pestis adherence in the lung. This hypothesis aligns with our prediction that there may be multiple factors involved in mediating adherence in the lung rather than a single Y. pestis adhesin.

**FIG 5:**
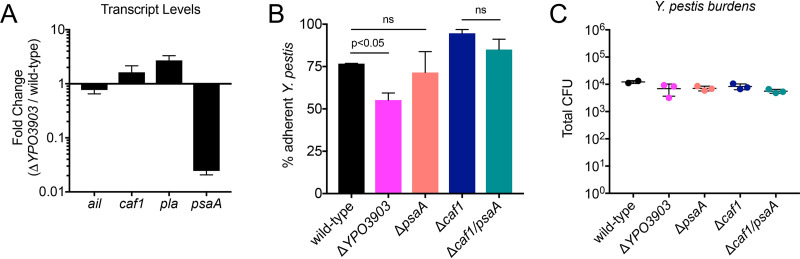
*YPO3903* regulates *psaA* levels, which contributes to Y. pestis adherence in the lung. (A) Fold change of transcript levels for *ail*, *caf1*, *pla*, and *psaA* detected by qRT-PCR in Δ*YPO3903* Y. pestis relative to the wild-type strain cultured at 37°C. (B and C) Mice were inoculated intranasally with 1 × 10^4^ CFU of the Y. pestis wild-type, Δ*YPO3903*, Δ*psaA*, Δ*caf1*, and Δ*caf1* Δ*psaA* strains, and bronchoalveolar lavage was performed at 2 hpi. CFU were enumerated in both the BALF and the lung. (B) Percent adherence was calculated for each strain by determining the proportion of Y. pestis in the lung compared to total CFU in both the BALF and the lung. (C) Total Y. pestis CFU enumerated in both the lung and the BALF. Data are representative of two independent experiments with 3 mice or replicates per group. Data are means and SD. *P* values were determined by one-way ANOVA with Tukey’s multiple correction. ns, not significant.

To test if YPO3903 regulates genes involved in adherence, we measured transcript levels of a small subset of Y. pestis genes previously implicated in *in vitro* adherence: *ail*, *caf1*, *pla*, and *psaA* (21, 23, 28). RNA was isolated from wild-type Y. pestis or Y. pestis Δ*YPO3903* cultures grown at 37°C. The RNA was converted to cDNA, and we performed quantitative reverse transcription-PCR (qRT-PCR) to measure *ail*, *caf1*, *pla*, and *psaA* transcript levels in each strain. While the *ail*, *caf1*, and *pla* transcript levels in Δ*YPO3903* were similar to wild-type levels (no more than a 2-fold change), the levels of *psaA* were reduced over 10-fold in Δ*YPO3903* Y. pestis relative to the wild-type strain (Fig. 5A). The reduction in *psaA* transcript levels correlated with a loss of PsaA protein levels in Δ*YPO3903* Y. pestis cultures compared to wild-type Y. pestis when strains were grown at 37°C (Fig. S3).

10.1128/mSphere.00715-20.3 FIG S3 PsaA protein levels are reduced in ΔYPO3903 Y. pestis relative to wild-type. Wild-type or Δ*YPO3903* Y. pestis cultures were grown for 6 h at 26°C or 37°C, and levels of PsaA were detected in whole-cell lysates via Western blotting. Prior to antibody probing, Ponceau S staining was used to determine loading in each lane (LC, loading control). Data are representative of two independent experiments. Download FIG S3, PDF file, 0.6 MB. Copyright © 2020 Eichelberger et al. 2020 Eichelberger et al. This content is distributed under the terms of the Creative Commons Attribution 4.0 International license.

If YPO3903 regulates levels only of *psaA*, we would predict that a *psaA* mutant would be as adherent as a *YPO3903* mutant *in vivo.* If YPO3903 regulates levels of *psaA* in addition to other genes involved in adherence, we would expect that the loss of *psaA* would have a minor effect on Y. pestis adherence *in vivo*. To test the role of *psaA* in Y. pestis adherence early during primary pneumonic plague, we inoculated mice intranasally with 1 × 10^4^ CFU wild-type Y. pestis, Y. pestis Δ*YPO3903*, or Y. pestis Δ*psaA.* At 2 hpi, bronchoalveolar lavage was performed, and the proportion of adherent Y. pestis bacteria for each strain was calculated. As seen previously, the *YPO3903* mutant had a significant defect in adherence relative to wild-type Y. pestis, with approximately 75% adherence calculated for wild-type Y. pestis and 55% adherence for Δ*YPO3903* Y. pestis (Fig. 5B). However, we observed only a minor loss of adherence in the *psaA* mutant, at ∼70% adherence, which was not statistically significant (Fig. 5B). The minor effect of *psaA* on adherence in the lung is also observed in the Δ*caf1* Y. pestis background (Fig. 5B). Each strain had a similar total Y. pestis burden recovered from the lung at 2 hpi of approximately 10^4^ CFU (Fig. 5C). These data support the hypothesis that PsaA contributes to some but not all of the Y. pestis adherence in the lung mediated by YPO3903. Thus, YPO3903 controls Y. pestis adherence likely by regulating levels of multiple adhesins at the transcriptional level, including *psaA*.

## DISCUSSION

Colonization of mucosal surfaces is a critical first step in the pathogenesis of *Yersinia* species. Following inhalation of Yersinia pestis, the bacteria colonize the lower airways, where unrestricted bacterial growth during an immunosuppressive phase of disease ultimately leads to severe inflammation and a fatal acute pneumonia (1, 2). Although several proteins have been proposed to mediate adherence *in vitro*, phenotypes for individual mutants are slight and the roles of these proteins in Y. pestis adherence in the lung have not been tested. The goal of our study was to identify genes important for Y. pestis adherence in the lung during primary pneumonic plague. We used Tn-seq to screen pools of wild-type and Δ*caf1* Y. pestis insertional mutants for defects in adherence in the lung.

For our screen, we used a lung lavage assay in which nonadherent bacteria are collected by bronchoalveolar lavage (41). Mutants with insertions in six genes were significantly enriched in both Y. pestis strain backgrounds: *YPO3904* and *iscR*, encoding transcriptional regulators; *clpX* and *tig*, encoding proteins that function as molecular chaperones; *rnc*, encoding an endoribonuclease; and *YPO3903*, encoding a hypothetical protein. While none of these genes are annotated to encode adhesins, each one encodes a protein involved in the regulation or assembly of macromolecules. The inactivation of these genes could result in the dysregulation of multiple adhesins. Overall, our Tn-seq results suggest that there may not be one predominant adhesin that Y. pestis utilizes for adherence in the lung. Identification of how each gene regulates the expression, assembly, or display of various surface proteins may reveal the various adhesins Y. pestis employs during infection.

A similar serial enrichment strategy was successfully applied to screen Caulobacter crescentus transposon mutants for defects in adherence *in vitro*, highlighting the utility of this approach in characterizing bacterial adherence (42). However, in our assay, the enrichment of mutants with transposon insertions in genes involved in gene regulation or protein display rather than adhesins also reflects a limitation of the Tn-seq approach: only single insertional mutants are created by transposon mutagenesis. If there is redundancy among adhesins, or if the loss of a single adhesin can be complemented by the presence of others, then we would not enrich for those particular mutants. Additionally, a unique issue with performing Tn-seq during primary pneumonic plague was identified previously: Y. pestis transposon mutants with a wild-type phenotype will transcomplement any avirulent mutants for growth in the lungs (1). The transcomplementation is dependent on the type III secretion system creating a permissive environment in the lungs to allow bacterial outgrowth of avirulent mutants. Since we were evaluating the ability of individual bacterial mutants to bind to the lung epithelium during the first 2 h after inoculation, and because the pCD1^−^ strain was just as adherent as wild-type Y. pestis, it is unlikely that transcomplementation affected the results of the screen in this study.

We created clean genetic deletions in our wild-type Y. pestis strain for five of the shared enriched mutants to determine their contribution to Y. pestis adherence. Of the deletion mutants tested for adherence *in vivo*, only the *YPO3903* mutant had a significant loss of adherence in the lung relative to wild-type Y. pestis (Fig. 4). Although Y. pestis Δ*YPO3904* did not have a statistically significant defect in adherence compared to wild-type Y. pestis, it was slightly less adherent in the lung. *YPO3904* encodes a transcriptional regulator with homology to HdfR, which is best characterized for its regulation of the flagellar master operon *flhDC* (43). However, HdfR has flagellum-independent activities, regulating expression of the glutamate synthase gene subunits *gltBD* in E. coli and colonization of the bladder during Proteus mirabilis infection (44, 45). HdfR may play a minor role in the regulation of genes required for Y. pestis adherence. We did not observe any significant loss of adherence for the *clpX*, *iscR*, and *tig* mutants compared to wild-type Y. pestis. These genes may play even subtler roles in regulating adherence that could be detectable if the genes were deleted in the more adherent Δ*caf1* strain background or when assayed during coinfection with other mutants, similar to the conditions of the Tn-seq screen.

*YPO3903* encodes a hypothetical protein, and we determined that YPO3903 has 74% identity to the E. coli protein YifE/MaoP. MaoP is conserved in *Enterobacteriaceae* and is involved in control of chromosome conformation and segregation, which can affect gene expression (40, 46). We hypothesized that YPO3903 may perform a similar function in Y. pestis and regulate expression or display of adhesins on the cell surface. Transcriptional analyses using quantitative PCR for a small subset of genes previously implicated in Y. pestis adherence *in vitro* revealed that transcript levels of *psaA* were about 10-fold lower in the Δ*YPO3903* strain than in the wild-type strain (Fig. 5A). The pH 6 antigen, encoded by *psaA*, increases adherence of Y. pestis to respiratory epithelial cells by binding phosphatidylcholine, but in the absence of the F1 antigen, a *psaA* mutant is just as adherent as wild-type Y. pestis (23, 47). Additionally, a Y. pestis *psaA* mutant has a minor virulence defect in the lung during primary pneumonic plague at 12 hpi that is not sustained as disease progresses (48). We observed a small but statistically insignificant loss of adherence in the lung for a *psaA* mutant in both the wild-type and Δ*caf1* Y. pestis strains (Fig. 5B). These data again suggest that multiple surface structures may be involved in Y. pestis adhesion in the lung and that YPO3903 likely regulates the expression of multiple genes involved in adherence, including *psaA*.

During our *in vivo* screen, we observed strong enrichment of mutants with a Δ*ail* colony morphology. This was surprising given our observations that an *ail* mutant was as adherent as wild-type Y. pestis early after inoculation (Fig. 1A). However, we observed a concurrent increase in the prevalence of the Δ*ail* colony morphology after *in vitro* enrichment, particularly in the Δ*caf1* background (Fig. 2D). Sequencing results reveal that *ail* mutants were significantly enriched in the Δ*caf1*, but not the wild-type, background (Table 1). Therefore, the enrichment of *ail* mutants *in vivo* could be due to enhanced growth or enrichment during the *in vitro* culture steps between passages in mice. Additionally, previous studies using *in vitro* assays demonstrated moderate defects in Y. pestis adherence to human epithelial cell lines at 37°C when *ail* was deleted individually, and these defects were enhanced when *ail* was deleted in combination with other surface protein-encoding genes, such as *pla* and *psaA* (16, 21). Taken together, these data suggest that *ail* may play a minor (if any) role in early Y. pestis adherence in the lung, which was magnified due to enhanced *in vitro* growth and multiple rounds of enrichment in our screen. The *ail* gene is one of the most highly expressed genes in Y. pestis at 37°C, and Ail constitutes a large fraction of the outer membrane (19, 49, 50). It is possible that a complete deletion of *ail* causes disruption of the outer membrane of Y. pestis. Outer membrane perturbations can nonspecifically alter serum sensitivity in E. coli (51). Therefore, the phenotypes we observed for a Y. pestis *ail* mutant *in vitro* (loss of adherence and serum sensitivity) may result from outer membrane perturbations due to the loss of Ail. However, molecular analyses of Ail revealed that different residues in the outer loops of the Ail protein contribute to Y. pestis adherence and serum resistance *in vitro* (52–54). Experiments to determine the role of these adherence-specific residues *in vivo* would provide a more sophisticated analysis of the contribution of Ail to Y. pestis adherence in the lung without the pleiotropic effects that result from the complete deletion of *ail*.

Adherence is not completely lost in a Y. pestis *YPO3903* mutant, suggesting that there are likely other proteins outside the control of YPO3903 that may play a role in adherence. Although we focused our analysis on the mutations in genes that were enriched in both the wild-type and Δ*caf1* backgrounds, there are many mutants with insertions in genes that were significantly enriched and unique to either wild-type or Δ*caf1* Y. pestis. We observed a relatively poor correlation between mutants enriched in the wild-type background and those in the *caf1* background (Fig. S2). These data imply that the presence of the F1 antigen dramatically alters display or availability of various surface structures for Y. pestis binding to host cells. Because Y. pestis produces the F1 antigen during primary pneumonic plague, mutants that were enriched in the wild-type background may be the most relevant to natural infection. There was significant enrichment of mutations in *micF*, which is a small regulatory RNA. In Y. pestis, MicF posttranscriptionally regulates levels of OmpF, an outer membrane protein (55). We also observed enrichment of mutations in *waaQ*, which encodes a lipopolysaccharide core biosynthesis protein (56). Since lipopolysaccharide (LPS) structure can affect the function of outer membrane proteins such as Ail, alterations in LPS resulting from a *waaQ* mutation may also affect adherence in Y. pestis (30, 57). Mutants with insertions in the gene *YPO3880* were also enriched in the wild-type Y. pestis background. YPO3880 is predicted to be an exported protein as part of a chaperone-usher system. The deletion of the operon containing this chaperone-usher system results in slight attenuation of a Y. pestis KIM strain following intranasal, but not intravenous, inoculation (29, 58). Characterizing the genes identified by our *in vivo* enrichment for nonadherent mutants, as well as further defining the role of *YPO3903* in regulating expression of Y. pestis genes involved in adherence, may reveal novel bacterial targets for inhibiting initial Y. pestis colonization of the lung during primary pneumonic plague.

## MATERIALS AND METHODS

### Bacterial strains and plasmids.

The fully virulent Yersinia pestis strain CO92 was obtained from the U.S. Army, Ft. Detrick, MD. The presence of pCD1 and the *pgm* locus was confirmed by PCR before use. Y. pestis was grown on brain heart infusion (BHI) agar (Difco Laboratories) at 26°C for 2 days. All bacterial strains used in this study are listed in Table S1.

10.1128/mSphere.00715-20.4 TABLE S1 Strains and primers used in this study. Barcode sequences for the index primers are highlighted in bold. Download Table S1, DOCX file, 0.03 MB. Copyright © 2020 Eichelberger et al. 2020 Eichelberger et al. This content is distributed under the terms of the Creative Commons Attribution 4.0 International license.

### Animals and animal infections.

Naive 6- to 8-week-old female C57BL/6J mice were obtained from Jackson Laboratories and housed in animal biosafety level 3 facilities at the University of North Carolina (UNC) at Chapel Hill prior to inoculation. All experiments involving mice were reviewed and approved by the Institutional Animal Care and Use Committee at UNC Chapel Hill under protocol number 15-022 or number 17-258. Y. pestis CO92 growth from a BHI agar plate was used to start liquid cultures in 2 ml BHI broth, which were grown for 12 h at 26°C. Cultures were then diluted 1:200 in 10 ml BHI broth supplemented with 2.5 mM CaCl_2_ and grown for 12 to 16 h at 37°C with constant shaking at 250 rpm. Mice were lightly anesthetized with 50 to 100 mg/kg ketamine and 5 to 10 mg/kg xylazine and then inoculated intranasally with a lethal dose of bacteria suspended in 20 μl sterile phosphate-buffered saline (PBS). Two hours after inoculation, mice were euthanized with an overdose of sodium pentobarbital.

### Determination of nonadherent Y. pestis.

Groups of mice were euthanized at 2 h postinoculation, and lungs were inflated via tracheal cannulation with 1 ml PBS. The PBS was then retracted to collect bronchoalveolar lavage fluid (BALF). This process was repeated until a total of 3 ml BALF was collected from each mouse. Lungs were then removed, placed in 1 ml PBS, and homogenized with a tissue homogenizer (Dremel). Serial dilutions of both the BALF and lung homogenate were plated on BHI agar to enumerate CFU. To calculate the percent nonadherent Y. pestis, the number of CFU in the BALF was divided by the sum of CFU in the lung homogenate and the BALF.

### Transposon mutagenesis.

The transposon mutant libraries were generated using the *Himar1*-based transposon system encoded on pPP47, which has been used previously for transposon mutagenesis in Yersinia pestis (1). E. coli S17 λ*pir* carrying pPP47 was mated with Y. pestis wild-type and Δ*caf1* strains twice. A 500-μl portion of liquid culture for each strain was washed with 1 ml 10 mM MgSO_4_, and then each Y. pestis strain was combined with E. coli in 50 μl 10 mM MgSO_4_, and this mixture was spotted on BHI agar. The wild-type Y. pestis transposon libraries are designated YP473Tn and the Δ*caf1* Y. pestis transposon libraries are designated YP475Tn. For YP473Tn and YP475Tn libraries, only one mating was performed for each strain. For YP473Tn3 and YP475Tn2 libraries, 4 individual matings were performed for each strain. Plates were incubated for 5 h at 26°C. A cotton swab was used to collect the resulting bacterial growth from each plate, which was resuspended in 1 ml PBS. For YP473Tn and YP475Tn, this was diluted 1:100 in PBS, and 250 μl was plated on 5 BHI agar plates containing kanamycin for Y. pestis selection with the transposon integrated into the genome and polymyxin B for E. coli counterselection. For YP473Tn3 and YP475Tn2, the resuspended mating was diluted 1:10 in PBS, and 200 μl was plated on 8 BHI agar plates with kanamycin and polymyxin B. Serial dilutions were also plated for each mating resuspension to determine the concentration of mutants in each mating. After 2 days incubation at 26°C, colonies were collected from the plates by flooding the top of the plate with BHI broth and dislodging colonies with a cell spreader. The medium was removed with a serological pipet and pooled in a 50-ml conical tube. Approximately 48,000 individual colonies for YP473Tn, ∼43,000 colonies for YP475Tn, ∼185,920 colonies for YP473Tn3, and ∼165,760 colonies for YP475Tn2 were collected. The colonies were resuspended in the BHI broth by repeated pipetting, and glycerol was added to a final concentration of 15%. Aliquots (500 μl) of each mating were kept at −80°C until use.

### Enrichment of transposon mutants *in vivo* and *in vitro*.

For the following protocol, the BHI agar or liquid broth used was supplemented with kanamycin (50 μg/ml) to maintain selection of transposon insertional mutants (kanamycin resistance). One frozen library aliquot from each transposon mutagenesis library (YP473Tn and YP473Tn3 for the wild-type background and YP475Tn and YP475Tn3 for the Δ*caf1* background) was thawed. YP473Tn was mixed with YP473Tn3 and YP475Tn was mixed with YP475Tn2. A 500-μl portion of the combined transposon libraries was added to separate 10-ml BHI broth cultures supplemented with 1 mM CaCl_2_ to generate the wild-type (WT) input (YP473Tn+YP473Tn3) and the Caf1 input (YP475Tn+YP475Tn2). The two libraries from the separate matings were combined to maximize diversity of insertional mutants in both input libraries. These cultures were incubated shaking at 37°C for 4 h. A 200-μl portion of each culture was added to 10 ml of fresh BHI broth supplemented with 1 mM CaCl_2_ and incubated with shaking for 15 h. The optical density at 620 nm (OD_620_) for each culture was determined, and 2 groups (designated WT *in vivo* group 1 and group 2) of 3 mice each were inoculated intranasally with 1 × 10^6^ CFU WT input, and 2 groups (designated Caf1 *in vivo* group 1 and group 2) of 3 mice each were inoculated intranasally with 5 × 10^6^ CFU Caf1 input. At the same time, 200 μl of the WT and Caf1 input cultures were added to 2 separate flasks of 10 ml BHI broth supplemented with 1 mM CaCl_2_ and were incubated with shaking at 37°C. Each flask was designated WT or Caf1 *in vitro* group 1 or group 2. At 2 hpi, mice were euthanized, lungs were inflated via tracheal cannulation with 1 ml ice-cold PBS, and the liquid was retracted to collect BALF. A total of 3 ml BALF was collected from each mouse. Lungs were then removed, placed in 1 ml PBS, and homogenized with a tissue homogenizer (Dremel). Serial dilutions of both the BALF and lung homogenate were made and plated on BHI agar to enumerate CFU. The remaining BALF (containing nonadherent Y. pestis mutants) was plated on BHI agar. The OD_620_ was determined for the *in vitro* cultures and 1 × 10^6^ CFU from each was diluted in 1 ml PBS and plated on BHI agar.

After 2 days of incubation at 26°C, the single colonies were collected from each plate. For each set of plates from a single mouse, BHI broth was poured on the surface of the agar plates. A plate spreader was used to dislodge the colonies from the surface of the agar into the medium, and this was collected with a serological pipet. The colony suspension was transferred to a 50-ml conical tube. This process was repeated for each set of plates from each mouse and each *in vitro* group. The colony suspensions for the 3 mice from group 1 were pooled, and the suspensions from group 2 were pooled for both the WT and Caf1 libraries. The colony suspensions from *in vitro* groups 1 and 2 were kept separate. A 500-μl portion of each pooled suspension was added to 500 μl of 50% glycerol and kept at −80°C. The strains generated from freezing aliquots of each library after enrichment are listed in Table S2. A 500-μl portion of each pooled bacterial suspension was added to 10 ml of fresh BHI broth with CaCl_2_ and incubated with shaking at 37°C for 4 h. Then, 200 μl of each culture was added to 10 ml of fresh BHI broth with CaCl_2_ and shaking at 37°C for 15 h. The process of inoculating groups of mice and the *in vitro* cultures and collecting mutants was repeated for a total of 4 rounds of enrichment *in vivo* and *in vitro*.

10.1128/mSphere.00715-20.5 TABLE S2 Strain name, description, sample name, and corresponding index primer used for each sample sequenced in the Tn-seq experiment. Download Table S2, DOCX file, 0.01 MB. Copyright © 2020 Eichelberger et al. 2020 Eichelberger et al. This content is distributed under the terms of the Creative Commons Attribution 4.0 International license.

### Preparation of bacterial genomes for sequencing.

Frozen aliquots of the WT and Caf1 libraries before, during, and after enrichment were thawed, and 500 μl of the stock was added to 10 ml BHI broth and grown with shaking at 37°C for 6 h. A 500-μl portion of this culture was added to fresh 10 ml BHI broth and grown shaking at 37°C for 12 h. Genomic DNA was isolated from 500 μl of culture using the Wizard Genomic DNA purification kit (Promega) following the manufacturer’s protocols. The names of all samples of DNA isolated from the corresponding pool of transposon mutants are listed in Table S2. Each DNA pellet was rehydrated overnight in 100 μl Tris-EDTA (TE) buffer at 4°C. An additional 100 μl TE buffer was added to each genomic DNA sample; then each sample was sheared using an EpiShear Probe sonicator (Active Motif) with 1 s on/1 s off for 30 s at 30% amplitude for 6 cycles, with 1 min of incubation on ice between cycles. Following sonication, each sample was purified using PCR purification with a QiaQuick kit (Qiagen) and eluted in 50 μl nuclease-free distilled H_2_O (dH_2_O). Samples were stored at −20°C until preparation for sequencing.

Each sheared DNA sample was diluted to 250 ng in a total volume of 50 μl nuclease-free dH_2_O. The KAPA Hyper Prep kit (KAPA Biosystems) was used to perform end repair, A-tailing, and adaptor ligation for each sample. Seven microliters of end repair and A-tailing buffer and 3 μl of end repair and A-tailing enzyme mix were added to the sheared DNA samples. The following thermocycler program was run: 30 min at 20°C, 30 min at 65°C, then hold at 4°C. Next, adaptor ligation was performed. The end repair and A-tailing product was added to 30 μl ligation buffer, 10 μl DNA ligase, 5 μl PCR water, and 5 μl of a 15 μM mix of adaptors A01 and A02. These samples were incubated at 20°C for 15 min. A postligation bead cleanup was performed using Agencourt AMPure XP beads, using a 0.8× bead selection according to the manufacturer’s protocols. The sample was eluted in 50 μl buffer EB (10 mM Tris-Cl, pH 8.5). A second bead cleanup was immediately performed with a 1× bead selection and elution of the DNA in 20 μl buffer EB. The samples were kept at −20°C until the PCRs.

PCR to amplify the transposon junctions was performed using the KAPA Robust 2G kit (KAPA Biosystems). The first PCR amplified sheared DNA sequences that had successfully ligated the adaptors. The 20-μl sample created with the KAPA Hyper Prep kit was added to 10 μl 5× buffer A, 5× enhancer, 10 mM deoxynucleoside triphosphates (dNTPs), 5 μM primer R1, 5 μM primer KAPA Prim 1, and 0.2 μl polymerase. The reaction was run in a thermocycler with the following program: 1 cycle of 98°C for 45 s; 10 cycles of 98°C for 15 s, 55°C for 30 s, and 72°C for 30 s; 1 cycle of 72°C for 1 min; then a hold at 4°C. A bead cleanup was performed for each sample with Agencourt beads, using a 1× bead selection according to the manufacturer’s protocols and eluting in 20 μl buffer EB. The second PCR further amplified the read fragments and added the sample-specific barcode sequence to allow pooling of the libraries for sequencing. This PCR was performed using KAPA HiFi *Taq* from the KAPA Hyper Prep kit (KAPA Biosystems). The 20-μl sample from the previous bead cleanup was added to 25 μl Ready Mix (2×), 5 μM index primer, and 5 μM primer KAPA Prim 1. The index primer used for each library is listed in Table S2. The reaction was run in a thermocycler with the following protocol: 1 cycle of 98°C for 45 s; 3 cycles of 98°C for 15 s, 63°C for 30 s, and 72°C for 15 s; 10 cycles of 98°C for 15 s, 65°C for 30 s, and 72°C for 30 s; 1 cycle of 72°C for 1 min; then a hold at 4°C. A bead cleanup was performed for each sample with Agencourt beads, using a 1× bead selection according to the manufacturer’s protocols and eluting in 20 μl buffer EB.

Each sample was analyzed on a Bioanalyzer (Agilent) to determine the molarity of the amplicons in the size range of 350 to 600 bp, which was the desired range of fragment lengths for sequencing. Each sample was diluted to 15 nM, and 5-μl portions of each sample was pooled, yielding a pooled sample volume of 90 μl. A double size selection was performed on the pooled sample using a 0.5 to 0.7× bead ratio with Agencourt beads following the manufacturer’s protocol. The double size selection further enriched the pooled sample for amplicons ranging from 200 to 800 bp. The sample was eluted in buffer EB, the concentration was determined by Qubit analysis, and the molarity was determined with a Bioanalyzer (Agilent). The sample was diluted to a final concentration of 30 nM in a 25-μl volume of buffer EB, which was sequenced using the Illumina MiSeq with 150× paired-end reads.

### Analysis of transposon mutants.

**(i) Read count and normalization.** The paired end reads generated 2 FASTQ files for each amplicon sequenced, named Read 1 (R1) and Read 2 (R2). Each read in the R2 FASTQ file was modified such that the four random nucleotides (NNNN in primer R1, which creates a random barcode to aid in identification of PCR duplicates), were removed and appended to the R2 identifier line. BWA (Burrows-Wheeler alignment tool) alignment (v0.7.17-r1194-dirty) to the Yersinia pestis CO92 full genome (33) was then performed on each paired-end set of FASTQ files and sorted using samtools (v1.8), after which duplicates were filtered using biobambam’s bammarkduplicate program (v2.0.33). This generated SAM (sequence alignment/map) files for each read.

Reads from the duplicate-filtered alignment were then inspected to find the insertion locations of the transposon. Most alignments allowed quick determination of the transposon insertion site, where a TA/AT dinucleotide was present followed by the transposon sequence. Other alignments resulted in a portion of the end sequence of the transposon aligning to the genome, thus obscuring the true start site. These states were suggested by the presence of soft clips adjacent to a match (denoted by an *n*S*n*M, *n*M*n*S, or *n*S*n*M*n*S pattern in the CIGAR string, where *n* denotes the number of nucleotides to which the designation of S, denoting a soft clip, or M, denoting a match, applies) of reads in the SAM file.

To confirm each suggested location, NCBI’s BLASTn (ncbi-blast v2.7.1+) program was used to determine the true start site of transposon insertion, where BLASTn was run with the transposon sequence against each of the suggested reads. BLAST, being a greedy aligner, would give results matching the entire transposon sequence if possible. For reads for which there was only an *n*M pattern in the CIGAR string, BLASTn would return a position such that the transposon insertion position complemented the start of the read as reported by BWA. For instances where a read’s CIGAR string contained a combination of soft clips and matches, any unaligned portion from BLASTn could be truly attributable to the Y. pestis genome. This allowed the resolution between BWA’s reported read and the exact location of the transposon insertion site in the genome. The number of unique insertions for each gene was quantified, and upper-quartile normalization was performed to give a read count for each gene.

### (ii) Calculating top hits.

The normalized read counts for each gene were averaged between the replicate samples, and this average was divided by the normalized read count for the input library and log_2_ transformed to give an output/input ratio for the following groups: WT *in vivo* 2 rounds, WT *in vivo* 3 rounds, WT *in vitro* 2 rounds, WT *in vitro* 3 rounds, Caf1 *in vivo* 2 rounds, Caf1 *in vivo* 3 rounds, Caf1 *in vitro* 2 rounds, and Caf1 *in vitro* 3 rounds. The mean and standard deviation of the output/input ratios for every gene within each individual group were calculated. Genes that were more than 2 standard deviations away from the mean in both *in vivo* 2 rounds or *in vivo* 3 were pursued for further analysis.

### Lambda red recombination.

Deletion of *clpX*, *iscR*, *tig*, *YPO3903*, and *YPO3904* was performed in Y. pestis wild-type and pCD1^−^ backgrounds using a modified form of lambda red recombination. Briefly, 500 bp upstream and 500 bp downstream sequences for the desired region to delete were amplified by PCR and combined in splicing by overhang extension (SOE) PCR with a Kan^r^ cassette flanked by FLP recombination target (FRT) sites for allelic replacement of the wild-type open reading frame (ORF) (59). The products were transformed into Y. pestis strains harboring pWL204, a plasmid containing the recombinase genes (10). Following successful recombination verified by PCR, the Kan^r^ cassette was resolved by the introduction of pSkippy, a Tet^s^ derivative of pFLP3 harboring an Amp^r^ cassette and *sacB* and carrying the FLP recombinase gene under the control of the *lac* promoter (60). Correct resolution of the Kan^r^ cassette, as well as presence of the virulence plasmids (for mutants in the Y. pestis wild-type background) and the *pgm* locus, was confirmed by PCR. The oligonucleotides used for lambda red recombination and the strains generated are listed in Table S1.

### Transcript quantification by qRT-PCR.

Two-milliliter portions of BHI broth cultures were inoculated with either wild-type or Δ*YPO3903* Y. pestis in the pCD1^−^ background and incubated with rolling overnight at 26°C. A 250-μl portion of each culture was added to 10 ml of fresh BHI broth supplemented with 1 mM CaCl_2_ and incubated with shaking for 6 h at 37°C. Total RNA was then purified from 1 ml of culture using the TRIzol reagent manufacturer’s protocol, treated with Turbo DNase (Ambion), and reverse transcribed with the iScript cDNA synthesis kit (Bio-Rad) according to the manufacturer’s instructions. cDNAs were used as the templates for amplification and detection of the Y. pestis genes *ail*, *caf1*, *pla*, and *psaA* with SYBR green dye (Bio-Rad) in an iCycler thermocycler (Bio-Rad). For each gene, the calculated threshold cycle (*C_T_*) was normalized to that of gyrase B (*gyrB*) from the same sample prior to calculation of the fold change using the ΔΔ*C_T_* method (61). Oligonucleotides used for qRT-PCR are listed in Table S1.

### Western blot analysis.

Two-milliliter portions of BHI broth cultures were inoculated with either wild-type or Δ*YPO3903* Y. pestis in the pCD1^−^ background and incubated with rolling overnight at 26°C. A 250-μl portion of each culture was added to 10 ml of fresh BHI broth supplemented with 1 mM CaCl_2_ and incubated with shaking for 6 h at 26°C or 37°C. Whole-cell lysates were prepared from cells at an OD_620_ of 2 that had been pelleted, washed once with ice-cold PBS, and resuspended in Laemmli buffer containing 5% β-mercaptoethanol. Samples were boiled for 10 min, and a portion corresponding to an OD_620_ of 0.2 was separated via SDS-PAGE and transferred to polyvinylidene difluoride (PVDF) membranes for Western blot analysis. Loading was qualitatively assessed by Ponceau S staining of the PVDF membrane. Anti-PsaA serum was used to probe for PsaA. Prior to use, the anti-PsaA serum was absorbed against E. coli lysates and used at a titer of 1:2,500 (62). Anti-IgG horseradish peroxidase (HRP)-conjugated secondary antibodies were used at a titer of 1:20,000.

### Statistical analysis.

Data were graphed and analyzed for statistical significance in Prism 7.0b. Data were analyzed by one-way analysis of variance (ANOVA) with Tukey’s multiple-comparison test or by Student's *t* test and are presented as means with standard deviations (SD). Details for statistical analyses, such as numbers of replicates, group numbers, the statistical test used, and the definition of statistical significance, are given in the figure legends.
