# Supplementary figures and images for: Tn-Seq Analysis Identifies Genes Important for Yersinia pestis Adherence during Primary Pneumonic Plague
Source: mSphere. 2020 Aug 5;5(4):e00715-20. doi: 10.1128/mSphere.00715-20 (PMC7407073; doi:10.1128/mSphere.00715-20)

Figure S1

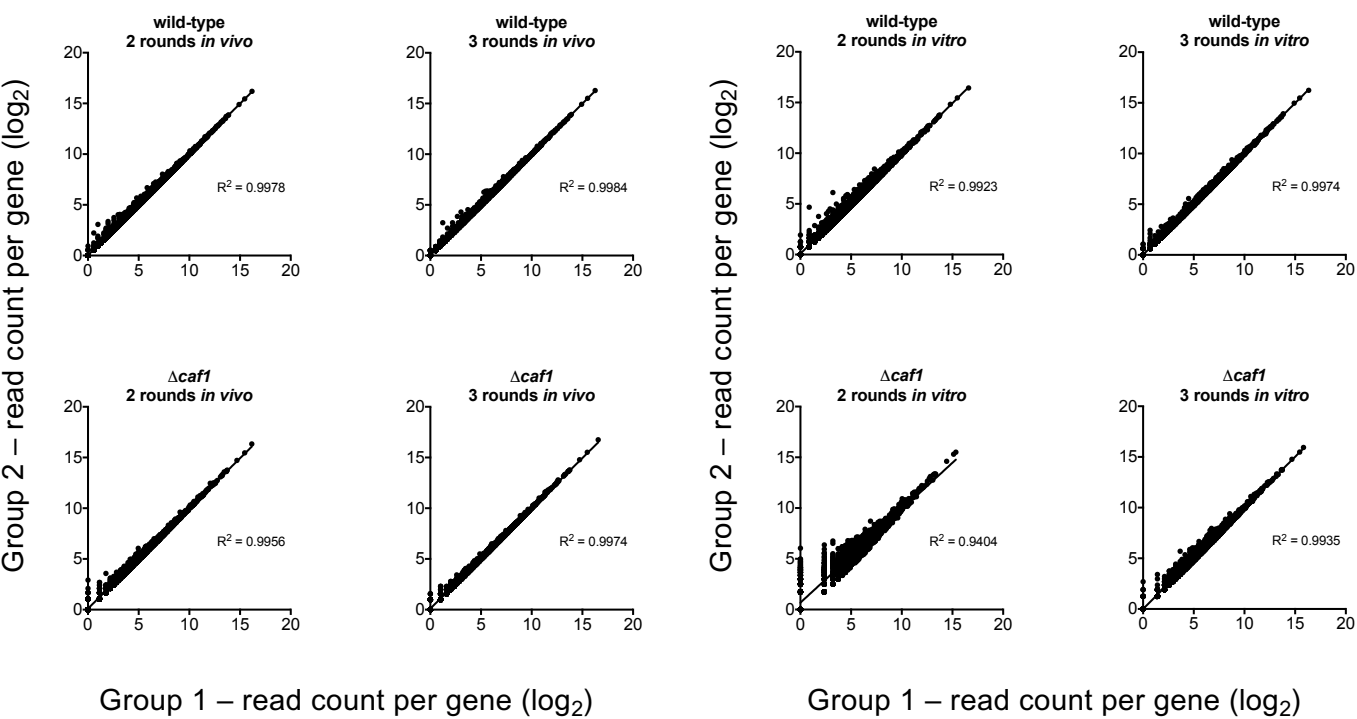

Supplement: FIG S1 [file mSphere.00715-20-sf001.pdf]

**Figure S2**

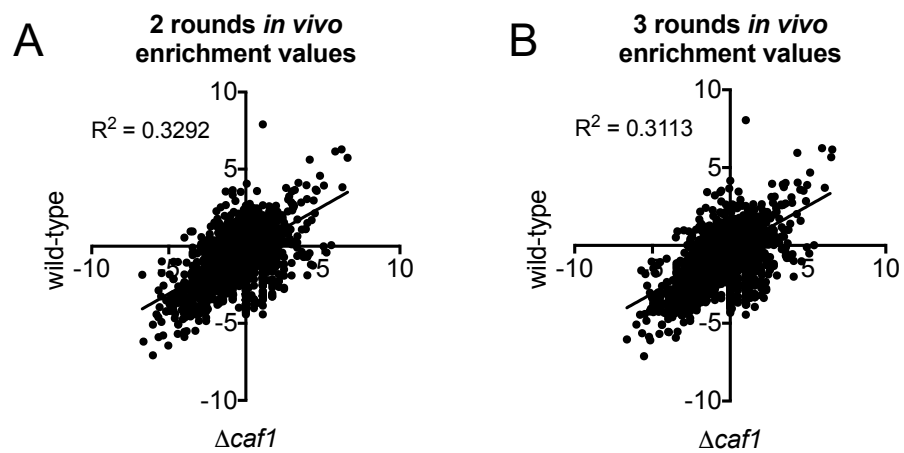

Supplement: FIG S2 [file mSphere.00715-20-sf002.pdf]

**Figure S3**

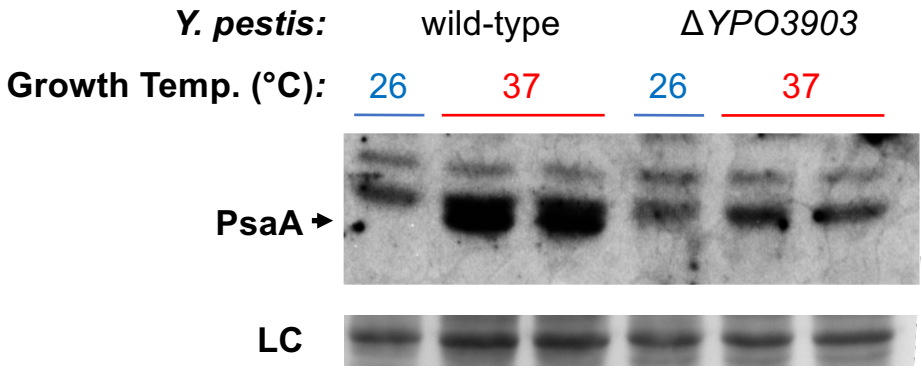

Supplement: FIG S3 [file mSphere.00715-20-sf003.pdf]
