# Supplementary material for: Tn-Seq Analysis Identifies Genes Important for Yersinia pestis Adherence during Primary Pneumonic Plague
Source: mSphere. 2020 Aug 5;5(4):e00715-20. doi: 10.1128/mSphere.00715-20 (PMC7407073; doi:10.1128/mSphere.00715-20)
Supplement: TABLE S1 [file mSphere.00715-20-st001.docx]

| **Strain Number** | **Strain description/genotype** | **Source/**  **Reference** |
| --- | --- | --- |
| YP03.5 | *Yersinia pestis* CO92 wild-type | Laboratory stock |
| YP229-1 | *Y. pestis* CO92 Δ*caf1* | (1) |
| YP314-1 | *Y. pestis* CO92 Δ*ail* (Δ*YPO2905*) | Laboratory stock |
| KE28 | *E. coli* S17 pPP47 | (2) |
| YP473Tn | *Y. pestis* CO92 *Himar1* insertional mutant pool | This study |
| YP473Tn3 | *Y. pestis* CO92 *Himar1* insertional mutant pool 2 | This study |
| YP475Tn | *Y. pestis* CO92 Δ*caf1* *Himar1* insertional mutant pool | This study |
| YP475Tn2 | *Y. pestis* CO92 Δ*caf1* *Himar1* insertional mutant pool 2 | This study |
| YP473Tn4 | *Y. pestis* CO92 YP473Tn Group 1 *in vivo* 2 rounds selection | This study |
| YP473Tn5 | *Y. pestis* CO92 YP473Tn Group 2 *in vivo* 2 rounds selection | This study |
| YP473Tn6 | *Y. pestis* CO92 YP473Tn Group 1 *in vitro* 2 rounds selection | This study |
| YP473Tn7 | *Y. pestis* CO92 YP473Tn Group 2 *in vitro* 2 rounds selection | This study |
| YP473Tn8 | *Y. pestis* CO92 YP473Tn Group 1 *in vivo* 3 rounds selection | This study |
| YP473Tn9 | *Y. pestis* CO92 YP473Tn Group 2 *in vivo* 3 rounds selection | This study |
| YP473Tn10 | *Y. pestis* CO92 YP473Tn Group 1 *in vitro* 3 rounds selection | This study |
| YP473Tn11 | *Y. pestis* CO92 YP473Tn Group 2 *in vitro* 3 rounds selection | This study |
| YP475Tn4 | *Y. pestis* CO92 YP475Tn Group 1 *in vivo* 2 rounds selection | This study |
| YP475Tn5 | *Y. pestis* CO92 YP475Tn Group 2 *in vivo* 2 rounds selection | This study |
| YP475Tn6 | *Y. pestis* CO92 YP475Tn Group 1 *in vitro* 2 rounds selection | This study |
| YP475Tn7 | *Y. pestis* CO92 YP475Tn Group 2 *in vitro* 2 rounds selection | This study |
| YP475Tn8 | *Y. pestis* CO92 YP475Tn Group 1 *in vivo* 3 rounds selection | This study |
| YP475Tn9 | *Y. pestis* CO92 YP475Tn Group 2 *in vivo* 3 rounds selection | This study |
| YP475Tn10 | *Y. pestis* CO92 YP475Tn Group 1 *in vitro* 3 rounds selection | This study |
| YP475Tn11 | *Y. pestis* CO92 YP475Tn Group 2 *in vitro* 3 rounds selection | This study |
| YP539-1 | *Y. pestis* CO92 Δ*clpX* | This study |
| YP540-1 | *Y. pestis* CO92 Δ*iscR* | This study |
| YP541 | *Y. pestis* CO92 Δ*tig* | This study |
| YP543 | *Y. pestis* CO92 Δ*YPO3903* | This study |
| YP544 | *Y. pestis* CO92 Δ*YPO3904* | This study |
| YP16 | *Y. pestis* CO92 Δ*psaA* | (3) |
| YP292 | *Y. pestis* CO92 Δ*caf1*Δ*psaA* | (1) |
| YP6 | *Y. pestis* CO92 pCD1^-^ | Laboratory stock |
| KE33 | *Y. pestis* CO92 pCD1^-^ Δ*ail* | Laboratory stock |
| KE70 | *Y. pestis* CO92 pCD1^-^ Δ*YPO3903* | This study |
| **Primer name** | **Sequence (5’-3’)** | **Source** |
| Adaptor A01 | AATGATACGGCGACCACCGAGATCTACACTAGATCGCNNWNNWNNACACTCTTTCCCTACACGACGCTCTTCCGATC*T | This study |
| Adaptor A02 | [Phos]GATCGGAAGAGCGTCGT*C*A*C | This study |
| pcr primer, R1 | GTGACTGGAGTTCAGACGTGTGCTCTTCCGATCTNNNNCAAAAGAATAGACCGAGATAGGGT | This study |
| KAPA  Prim 1 | AATGATACGGCGACCACCGA | This study |
| pcr primer, index 1 | CAAGCAGAAGACGGCATACGAGAT**CGTGAT**GTGACTGGAGTTCAGACGTGTGCTC | This study |
| pcr primer, index 2 | CAAGCAGAAGACGGCATACGAGAT**ACATCG**GTGACTGGAGTTCAGACGTGTGCTC | This study |
| pcr primer, index 3 | CAAGCAGAAGACGGCATACGAGAT**GCCTAA**GTGACTGGAGTTCAGACGTGTGCTC | This study |
| pcr primer, index 4 | CAAGCAGAAGACGGCATACGAGAT**TGGTCA**GTGACTGGAGTTCAGACGTGTGCTC | This study |
| pcr primer, index 5 | CAAGCAGAAGACGGCATACGAGAT**CACTGT**GTGACTGGAGTTCAGACGTGTGCTC | This study |
| pcr primer, index 6 | CAAGCAGAAGACGGCATACGAGAT**ATTGGC**GTGACTGGAGTTCAGACGTGTGCTC | This study |
| pcr primer, index 7 | CAAGCAGAAGACGGCATACGAGAT**GATCTG**GTGACTGGAGTTCAGACGTGTGCTC | This study |
| pcr primer, index 8 | CAAGCAGAAGACGGCATACGAGAT**TCAAGT**GTGACTGGAGTTCAGACGTGTGCTC | This study |
| pcr primer, index 9 | CAAGCAGAAGACGGCATACGAGAT**CTGATC**GTGACTGGAGTTCAGACGTGTGCTC | This study |
| pcr primer, index 10 | CAAGCAGAAGACGGCATACGAGAT**AAGCTA**GTGACTGGAGTTCAGACGTGTGCTC | This study |
| pcr primer, index 11 | CAAGCAGAAGACGGCATACGAGAT**GTAGCC**GTGACTGGAGTTCAGACGTGTGCTC | This study |
| pcr primer, index 12 | CAAGCAGAAGACGGCATACGAGAT**TACAAG**GTGACTGGAGTTCAGACGTGTGCTC | This study |
| pcr primer, index 13 | CAAGCAGAAGACGGCATACGAGAT**TATGGA**GTGACTGGAGTTCAGACGTGTGCTC | This study |
| pcr primer, index 14 | CAAGCAGAAGACGGCATACGAGAT**TAGTAC**GTGACTGGAGTTCAGACGTGTGCTC | This study |
| pcr primer, index 15 | CAAGCAGAAGACGGCATACGAGAT**TACTGT**GTGACTGGAGTTCAGACGTGTGCTC | This study |
| pcr primer, index 16 | CAAGCAGAAGACGGCATACGAGAT**CATGAG**GTGACTGGAGTTCAGACGTGTGCTC | This study |
| pcr primer, index 17 | CAAGCAGAAGACGGCATACGAGAT**TATCGT**GTGACTGGAGTTCAGACGTGTGCTC | This study |
| pcr primer, index 18 | CAAGCAGAAGACGGCATACGAGAT**TCTGCA**GTGACTGGAGTTCAGACGTGTGCTC | This study |
| clpX up F | GCTCGCCACTGTCAGAAAGC | This study |
| clpX up  R + P1 | GAAGCAGCTCCAGCCTACACTTAACCAGATTGTCCAGGCAG | This study |
| clpX down F + P4 | GGTCGACGGATCCCCGGAATCATCAGTAAACCTCTTCTTTA | This study |
| clpX down R | GGGGCAAGCATGTTCAATGGG | This study |
| iscR up F | CACGCCGATCTCATTATTCACATGCATG | This study |
| iscR up  R + P1 | GAAGCAGCTCCAGCCTACACTAAGCAGAATTTGCGGAATTTTA | This study |
| iscR  down F + P4 | GGTCGACGGATCCCCGGAATCATAGCTAAAGTTACCTGTT | This study |
| iscR down R | TCCCCGCCAATCCTGAATATAGCTCC | This study |
| tig up F | CACCAGAGCCATGTTAGGTGC | This study |
| tig up R + P1 | GAAGCAGCTCCAGCCTACACTGTTTTTCAGCGCATGATGC | This study |
| tig down  F + P4 | GGTCGACGGATCCCCGGAATCATCTTGTTACCTCAAAAAAA | This study |
| tig down R | GCAGGCAGTGTTCACGCGCTGGCG | This study |
| YPO3903 up F | GCACCTGAGGATGCCGCGTTTC | This study |
| YPO3903 up R + P1 | GAAGCAGCTCCAGCCTACACTCTCCCTGCGTACTTGAAGCTGCAGG | This study |
| YPO3903 down F + P4 | GGTCGACGGATCCCCGGAATCATCTGACACGCTCCTAATTCT | This study |
| YPO3903 down R | CACCAGTGAGTTTCTCAGCGC | This study |
| YPO3904  up F | CGAATCCCGATGAGCTTACAT | This study |
| YPO3904  up R + P1 | GAAGCAGCTCCAGCCTACACCACACCATCTCCAATTTTTTTTA | This study |
| YPO3904 down F + P4 | GGTCGACGGATCCCCGGAATTTACACGGGAGTAAGTGGCGATG | This study |
| YPO3904 down R | CGTGTTCCTGGGGGTGGGCGATT | This study |
| ail qRT F | GTCAACAGATTCCTCGAACCC | This study |
| ail qRT R | AAGTCGTGTCAAGGAAGATGG | This study |
| caf1 qRT F | TTAACTTTACAGATGCCGCGG | This study |
| caf1 qRT R | CTCACCGTTTACCTTAGGAGA | This study |
| pla qRT F | GGCTTCCGGGTCAGGTAATAT | This study |
| pla qRT R | CCTGGAGTAACCAGCCTTTCA | This study |
| psaA qRT F | TTTTTGCGGGTAAACAGCCGG | This study |
| psaA qRT R | AACCAACATAGTCACCATCGG | This study |
| gyrB qRT F | ATCCGTTGTTAACGCCCTGTCTGA | (4) |
| gyrB qRT R | ACAGTAGTCCCGGTTTGCTCAGTT | (4) |

1. **Weening EH**, **Cathelyn JS**, **Kaufman G**, **Lawrenz MB**, **Price P**, **Goldman WE**, **Miller VL**. 2011. The dependence of the *Yersinia pestis* capsule on pathogenesis is influenced by the mouse background. Infect Immun **79**:644–652.

2. **Price PA**, **Jin J**, **Goldman WE**. 2012. Pulmonary infection by *Yersinia pestis* rapidly establishes a permissive environment for microbial proliferation. Proc Natl Acad Sci USA **109**:3083–3088.

3. **Cathelyn JS**, **Crosby SD**, **Lathem WW**, **Goldman WE**, **Miller VL**. 2006. RovA, a global regulator of *Yersinia pestis*, specifically required for bubonic plague. Proc Natl Acad Sci USA **103**:13514–13519.

4. **Pechous RD**, **Broberg CA**, **Stasulli NM**, **Miller VL**, **Goldman WE**. 2015. *In Vivo* transcriptional profiling of *Yersinia pestis* reveals a novel bacterial mediator of pulmonary inflammation. mBio **6**:e02302–14–11.
