# Supplementary material for: Tn-Seq Analysis Identifies Genes Important for Yersinia pestis Adherence during Primary Pneumonic Plague
Source: mSphere. 2020 Aug 5;5(4):e00715-20. doi: 10.1128/mSphere.00715-20 (PMC7407073; doi:10.1128/mSphere.00715-20)
Supplement: TABLE S2 [file mSphere.00715-20-st002.docx]

| **Strain name** | **Description** | **Sample name** | **Index primer** |
| --- | --- | --- | --- |
| YP473Tn + YP473Tn3 | *Y. pestis* CO92 YP473Tn input library | WT input | pcr primer, index 1 |
| YP473Tn4 | *Y. pestis* CO92 YP473Tn Group 1 *in vivo* 2 rounds enrichment | WT in vivo 1 | pcr primer, index 2 |
| YP473Tn5 | *Y. pestis* CO92 YP473Tn Group 2 *in vivo* 2 rounds enrichment | WT in vivo 2 | pcr primer, index 3 |
| YP473Tn8 | *Y. pestis* CO92 YP473Tn Group 1 *in vivo* 3 rounds enrichment | WT in vivo 3 | pcr primer, index 4 |
| YP473Tn9 | *Y. pestis* CO92 YP473Tn Group 2 *in vivo* 3 rounds enrichment | WT in vivo 4 | pcr primer, index 5 |
| YP473Tn6 | *Y. pestis* CO92 YP473Tn Group 1 *in vitro* 2 rounds enrichment | WT in vitro 1 | pcr primer, index 6 |
| YP473Tn7 | *Y. pestis* CO92 YP473Tn Group 2 *in vitro* 2 rounds enrichment | WT in vitro 2 | pcr primer, index 7 |
| YP473Tn10 | *Y. pestis* CO92 YP473Tn Group 1 *in vitro* 3 rounds enrichment | WT in vitro 3 | pcr primer, index 8 |
| YP473Tn11 | *Y. pestis* CO92 YP473Tn Group 2 *in vitro* 3 rounds enrichment | WT in vitro 4 | pcr primer, index 9 |
| YP475Tn + YP475Tn2 | *Y. pestis* CO92 YP475Tn input library | Caf1 input | pcr primer, index 10 |
| YP475Tn4 | *Y. pestis* CO92 YP475Tn Group 1 *in vivo* 2 rounds enrichment | Caf1 in vivo 1 | pcr primer, index 11 |
| YP475Tn5 | *Y. pestis* CO92 YP475Tn Group 2 *in vivo* 2 rounds enrichment | Caf1 in vivo 2 | pcr primer, index 12 |
| YP475Tn8 | *Y. pestis* CO92 YP475Tn Group 1 *in vivo* 3 rounds enrichment | Caf1 in vivo 3 | pcr primer, index 13 |
| YP475Tn9 | *Y. pestis* CO92 YP475Tn Group 2 *in vivo* 3 rounds enrichment | Caf1 in vivo 4 | pcr primer, index 14 |
| YP475Tn6 | *Y. pestis* CO92 YP475Tn Group 1 *in vitro* 2 rounds enrichment | Caf1 in vitro 1 | pcr primer, index 15 |
| YP475Tn7 | *Y. pestis* CO92 YP475Tn Group 2 *in vitro* 2 rounds enrichment | Caf1 in vitro 2 | pcr primer, index 16 |
| YP475Tn10 | *Y. pestis* CO92 YP475Tn Group 1 *in vitro* 3 rounds enrichment | Caf1 in vitro 3 | pcr primer, index 17 |
| YP475Tn11 | *Y. pestis* CO92 YP475Tn Group 2 *in vitro* 3 rounds enrichment | Caf1 in vitro 4 | pcr primer, index 18 |
